# Knowledge, Truth, and Social Reality: An Introductory Note on Qualitative Research

**DOI:** 10.4103/0970-0218.69249

**Published:** 2010-07

**Authors:** N Nakkeeran

**Affiliations:** Indian Institute of Public Health, Gandhinagar, Ahmedabad, Gujarat, India

## Introduction

Social sciences have contributed significantly to health research both in terms of specific content and in terms of methodology. Individual social disciplines with their respective characteristic research procedures have equipped health research with a greater variety and possibilities of enquiry. Social sciences’ contribution through qualitative research is of particular importance. It is true that not all social science researches are qualitative in nature and not all the qualitative researches done are by social scientists. However, it is equally true that qualitative research methods have evolved as an integral feature of social sciences along with the way these disciplines understood reality and what according to them constituted knowledge. In other words, a basic understanding of social sciences, how they looked at reality, and their logic of knowledge synthesis and theory building could be a useful starting point to appreciate the nature and strengths of qualitative methods.

## Forms of Knowledge

Knowledge refers to an expertise or skills possessed or acquired by an individual. Knowledge refers to an understanding of the world around us that helps us to lead our life as a member of society. It helps to predict events and hence to mitigate the suffering or enhance the well-being of individuals and groups. We commonly understand that acquisition of knowledge is possible through two fundamental means: by experience (empirical) and reasoning (logical). The former includes the knowledge we gain through sensory perceptions and the latter includes logic and mathematical knowledge. However, in practice, we gain knowledge through processes that are combinations of experience and reasoning.

Knowledge often gets tagged with a connotation of truth. Accordingly, if something has to be considered as knowledge then it has to be true. Only if it is true it qualifies as a form of knowledge otherwise it is not considered as part of knowledge. I believe that Earth is spherical in shape and it revolves around the Sun. I think that infections are caused by germs. These statements are expressions of knowledge because they are truths.

However, in practical life not all forms of knowledge can be subjected to the test of truth and falsehood. There are forms of knowledge which cannot be subjected to this test yet are very much essential to lead our life. As a member of a society we learn the morals that govern our life. We learn to lead our life as per these norms, values, opinions, preferences, etc. There is a possibility that some individuals are more knowledgeable than others with regard to these values and norms. Knowledge of these values facilitates the collective life. This knowledge cannot be tested in the dimension of truth and falsehood but only in the dimension of good or bad. This domain of knowledge could be called as morals. Most decisions we make in our day-to-day life are governed by this domain of knowledge. Our decisions on marriage, at what age to get married, how many children to have, preferring a male child, kind of food we eat, our life-style, feeding and rearing a child, care seeking and care giving, allocating and distributing funds in a family or even in organizations, electoral decisions, so and so forth are decided based on this form of knowledge rather than through an exhaustive search for truth. Terming this form of knowledge as practical knowledge, Immanuel Kant (1864–1920) distinguished it from theoretical knowledge.(1)

Similarly, we have other forms of knowledge such as art (to differentiate between different musical ragas), esthetics (appreciating a painting), and religious knowledge which cannot be tested in the dimension of truth and falsehood.

## Science - Natural and Social Sciences

Science refers to a particular form of knowledge, which could be relied on to gain a more dependable, correct, or true understanding of the world, and how the world works. It also refers to a search for knowledge using a set of systematic principles such as objectivity and measurability which are universally accepted. It is distinct from an outlook based on religion, faith, or belief.

Having said this, we have to understand the fact that there are a number of academic disciplines ranging from mathematics and physics to psychology and sociology all grouped as sciences. They can be arranged in a hierarchy in terms of degree of objectivity, certainty, and universality of their explanations. Theories in sciences such as mathematics and physics can claim to a very high degree of certainty, objectivity, and universality as they are to great extent independent of human experience. To a significant level, the same might be true for explanations in disciplines of anatomy and physiology. However, when we come to social science disciplines such as sociology, anthropology, or psychology, their explanations cannot and do not claim for higher degree of objectivity, certainty, or universality. All sciences aspire to understand reality and/or attempt to explain how the world works. Although this can be common for all the disciplines, social sciences differ enormously from natural sciences in terms of the way they look at reality, part of the reality they choose to study, and the kind of problems they choose to address.

## Human Consciousness and Subjectivity

The reality that natural sciences want to study is devoid of human aspects such as conscience and subjectivity. On the other hand, social sciences involve study of human consciousness and subjectivity at the level of both observed and the observer. It includes the study of belief, values, intentions, and meanings attributed to human actions within a culture. The logic of explanation for an apple falling from a tree might hold good for a man falling from the 14^th^ floor as well but that is obviously not a sufficient explanation and that is not the explanation we talk about in social sciences. Such a “reality” that is being studied by social sciences is not transparently available for an exterior gaze, but has to be elicited from within, hence the possibility of interpretation as well as social construction of reality.(2)

## Disconnect between Process and Object of Study

Natural sciences assume a necessary disconnect between the process of studying and the object that is being studied. The reality that is studied or the phenomenon that is observed is considered external to the process of studying. A falling apple or a swinging pendulum is “out there” as an objective reality distinct from the observer. The “reality” that is being observed is independent of the observer and vice versa. They are called objects of study.

The “reality” that social sciences want to study is not like falling apples or a swinging pendulum, but are human beings and human mind. The reality is not “out there.”(3) We prefer to use the word subject rather than object. An object is passive and inert with respect to the process happening around it. A subject by definition has subjectivity, consciousness, or an inner cognitive process. A subject connotes an active reception of any external process, with an agency. In other words, there is no clear disjunction between the process of study and the “subject” of study. There is every possibility of the observed being influenced by the presence of an observer and vice versa.

## Deductive and Inductive Logic

**Figure 1:**
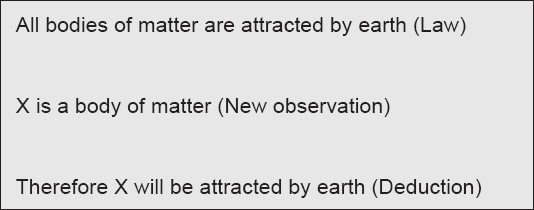
Deductive logic

For the reasons cited above, the approaches and methods used are vastly different between natural and social sciences. In natural sciences, a new observation is often explained through preexisting axioms, laws, or theories. In other words we deduce our explanations from a known fact. Figure 1 indicates one such typical deduction.

**Figure 2:**
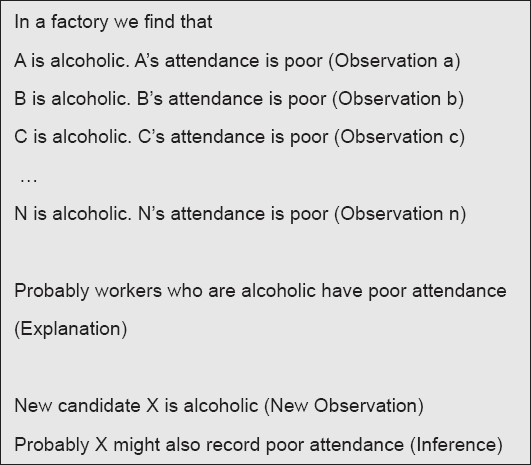
Inductive logic

In social sciences however we do not have any such invariant laws or theories that hold good over time and across all societies. Hence, we cannot fall back on any such laws to deduce an explanation for a new observation. Instead we try to collect a large number of similar observations in which a particular explanation is holding good and hence arrive at an explanation which would probably hold good for newer observations. In other words, we continuously induct newer observations to a body of evolving explanation to arrive at a relatively more robust explanation for a set of related observations [Figure 2]. However, this explanation can never qualify as a theory or a law as there is always a possibility that a new observation can disprove the explanation that was developed. This compels us to alter or refine the explanation so as to incorporate this particular new observation and so on.

Over the years of their existence, through induction, social sciences have arrived at explanations of varying degrees of robustness for different social phenomena. Hence quite a number of observations are explicable using these explanations. However, as the reality studied is dynamic and varying across societies and time, there is always a need for increasingly refined, altered, or nuanced explanations.

## Conclusion

In the foregoing pages, we looked at what constitute knowledge and that there could be different forms of knowledge. Social science is often concerned with particular forms of knowledge which cannot be subjected to the test of truth and falsehood. This is because social science is most often concerned with human behaviors and decisions which are directed by normative considerations of good/bad and right/wrong rather than in the dimension of true/false. Many problems in public health too involve understanding and dealing with such behaviors or decisions. Social sciences research in health, and thus aim more to understand such behaviors and decisions in practical life with implications on health.
